# Profiling of differentially expressed microRNAs in arrhythmogenic right ventricular cardiomyopathy

**DOI:** 10.1038/srep28101

**Published:** 2016-06-16

**Authors:** Hongliang Zhang, Shenghua Liu, Tianwei Dong, Jun Yang, Yuanyuan Xie, Yike Wu, Kang Kang, Shengshou Hu, Deming Gou, Yingjie Wei

**Affiliations:** 1 State Key Laboratory of Cardiovascular Disease, Fuwai Hospital, National Center for Cardiovascular Disease, Chinese Academy of Medical Sciences and Peking Union Medical College, Beijing 100037, People’s Republic of China; 2 Department of Cardiology, the First Affiliated Hospital of Jiamusi University, Jiamusi, Heilongjiang, 154002, People’s Republic of China; 3 Shenzhen Key Laboratory of Microbial Genetic Engineering, Shenzhen Key Laboratory of Marine Bioresource and Eco-environmental Science, College of Life Sciences, Shenzhen University, Shenzhen, Guangdong, 518060, People’s Republic of China; 4 Department of Physiology, Shenzhen University Health Science Center, Shenzhen, Guangdong 518060, People’s Republic of China

## Abstract

Arrhythmogenic right ventricular cardiomyopathy (ARVC) is a kind of primary cardiomyopathy characterized by the fibro-fatty replacement of right ventricular myocardium. Currently, myocardial microRNAs have been reported to play critical role in the pathophysiology of cardiovascular pathophysiology. So far, the profiling of microRNAs in ARVC has not been described. In this study, we applied S-Poly (T) Plus method to investigate the expression profile of microRNAs in 24 ARVC patients heart samples. The tissue levels of 1078 human microRNAs were assessed and were compared with levels in a group of 24 healthy controls. Analysis of the area under the receiver operating characteristic curve (ROC) supported the 21 validated microRNAs to be miRNA signatures of ARVC, eleven microRNAs were significantly increased in ARVC heart tissues and ten microRNAs were significantly decreased. After functional enrichment analysis, miR-21-5p and miR-135b were correlated with Wnt and Hippo pathway, which might involve in the molecular pathophysiology of ARVC. Overall, our data suggested that myocardial microRNAs were involved in the pathophysiology of ARVC, miR-21-5p and miR-135b were significantly associated with both the myocardium adipose and fibrosis, which was a potential disease pathway for ARVC and might to be useful as therapeutic targets for ARVC.

Arrhythmogenic right ventricular cardiomyopathy (ARVC), a typically autosomal dominant heart muscle disease coupled with ventricular enlargement, dysfunction and lethal arrhythmias, is the primary reason of sudden death in young people and athletes, the estimated prevalence of ARVC in the general population is about 1/2000–5000, the hallmark pathogenesis of ARVC is the myocardium tissue being replaced by fibro-fatty^1^,^2^,^3^. During the last two decades, the genetic analysis of ARVC fostered the view that it is a desmosome dysfunction disease. Several causative desmosome mutations of genes have been discovered in ARVC, including plakoglobin (PG), desmoplakin (DSP), plakophilin-2 (PKP2), desmoglein-2 (DSG2) and desmocollin-2 (DSC2)^1^,^4^. Moreover, extra desmosome genes has also been identified, such as the transforming growth factor-β3 gene (TGF-β3)^5^, connexin43 (Cx43)^6^,^7^and TMEM43 8.

Wnt/β-catenin pathway was well known for its pathogenic role as a key regulator of myogenesis versus adipogenesis. Previous studies showed that down-regulation of DSP expression led to the release of PG from the desmosomes. PG could change its location to nucleus and compete with β-catenin to suppress the canonical Wnt/β-catenin pathway; thereby enhancing adipogenesis was induced by nuclear transcription factors PPARγ and C/EBP-a^9^. It is recognized that there was a crosstalk between Wnt/β-catenin and Hippo/YAP signaling pathways^10^. Recently, a novel molecule mechanism of ARVC was identified. Desmosome disruption could perturb the ancient pathway, Hippo/YAP pathway, which was central to regulation of cellular proliferation and has been considered to control cardiomyocyte proliferation and heart size^11^, Hippo pathway activation suppressed the canonical Wnt signaling pathway and enhanced adipogenesis in ARVC^12^.

MicroRNAs are a class of small noncoding RNAs (~20 nucleotides), which regulate the expression of protein-coding genes post-transcriptionally through interacting with the 3′-untranslated region (3′-UTR) of their target mRNAs^13^,^14^,^15^. To date, more than 2000 human microRNAs have been identified^16^. The current research manifested that microRNAs were play widespread roles in cardiovascular pathologies^17^,^18^. And numerous studies indicated that microRNAs have been orchestrated in regulation of heart development and function^19^,^20^. The myocardial microRNAs play essential roles in complicated cardiovascular disease, including acute myocardial infarction, hypertrophic cardiomyopathy, heart failure, angiogenesis, atherosclerosis, arrhythmia and cardiomyopathy^21^,^22^,^23^. For example, the myocardial-specific microRNAs, miR-1, miR-133a, miR-133b, and miR-208a were significantly varied among cardiomyopathies, in muscle and cardiomyocytes, miR-1 and miR-133 is drawn into the same bicistronic unit^24^, and miR-208 is locked in the introns which encode the α-myosin heavy chain and β-myosin heavy chain^25^. Hua *et al*. revealed that miR-1, miR-133a and miR-208 had an impaired expression in hearts underwent cardiac hypertrophy and stress-dependent cardiomyocytes growth in different murine models of hypertrophic cardiomyopathy^26^. However, the connection between ARVC and microRNAs is still largely unknown.

In this study, we hypothesized that miRNAs may contribute to modulate fibro-fatty formation and involve in the pathological mechanism of ARVC. We assessed the myocardial level of 1078 human microRNAs in 24 ARVC patients and 24 healthy controls. The differentially expressed microRNAs were further analyzed, we found two significantly altered microRNAs candidates which might contribute to ARVC. We expect that these findings could provide a basis for further research of the molecular mechanism underlying the development of ARVC.

## Results

### Clinical characteristics of ARVC patients

Human right ventricle samples were obtained from 24 unrelated end-stage ARVC patients who underwent heart transplantation in Fuwai hospital (Beijing, China) between 2005 and 2014. All heart tissues were diagnosed by myocardial histology to identify replacement of fibro-fatty, each patient underwent clinical evaluation consisting of a detailed personal/family history, physical examination, 12-lead electrocardiogram (ECG), 24 h ECG Holter monitoring, transthoracic echocardiograph, and cardiac magnetic resonance (CMR). The detailed clinical characteristics are summarized in Table 1.

### Expression profiling of microRNAs in ARVC pooled samples

**Figure 1:**
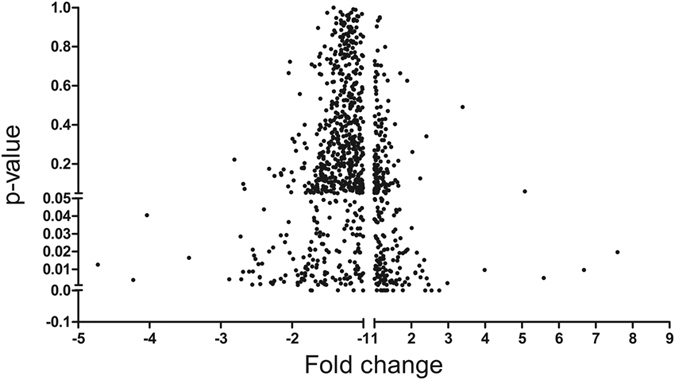
microRNAs differentially expressed in ARVC pool and control hearts pool were shown in valcano plot, significantly up-regulated and down-regulated microRNAs are depicted respectively.

**Figure 2:**
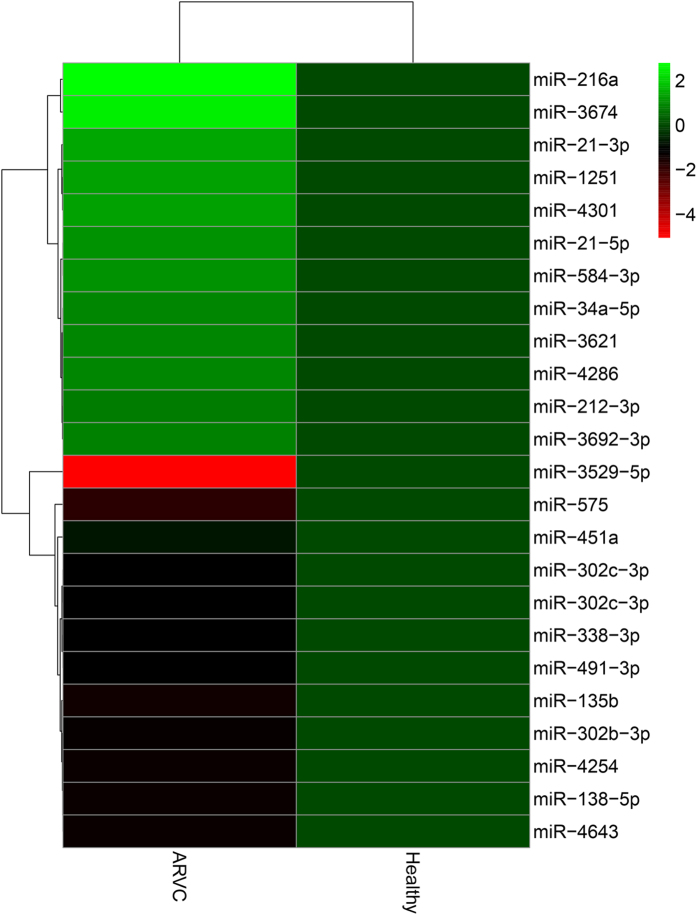
Heat map analysis of identified microRNAs based on screening outcomes, the microRNAs with a p-value < 0.001 are shown, Green: up-regulated, red: down-regulated.

Expression profiles of total 1078 microRNAs were investigated in RNA mixtures of ARVC heart samples and the normal control samples by S-Poly(T) Plus method^27^. Each miRNA was assayed in triplicates on 96-well plates by qRT-PCR and the microRNAs with cycle threshold (Ct) value more than 35 were excluded. The relationship between miRNA fold changes and corresponding statistical difference were presented in a volcano Plot (Fig. 1). The miRNA expression levels were considered significantly different with at least a 1.5 fold-change in RAVC group compared to control group. According to these criteria, a total of 24 significantly varied microRNAs were identified, with 12 up-regulated microRNAs (miR-21-3p, miR-21-5p, miR-34a-5p, miR-212-3p, miR-216a, miR-584-3p, miR-1251, miR-3621, miR-3674, miR-3692-3p, miR-4286, miR-4301) and 12 down-regulated microRNAs (miR-135b, miR-138-5p, miR-193b-3p, miR-302b-3p, miR-302c-3p, miR-338-3p, miR-451a, miR-491-3p, miR-575, miR-3529-5P, miR-4254, miR-4643) (Fig. 2).

### Validation each of candidate microRNAs

**Figure 3:**
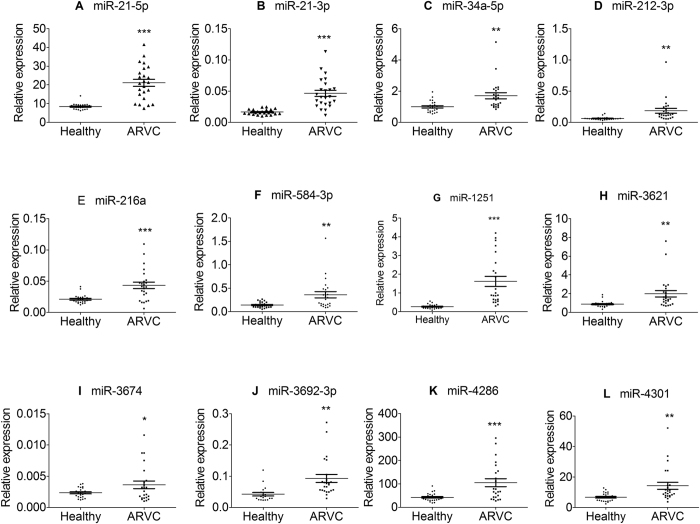
Differentially expressed 12 up-regulated microRNAs in the ARVC patients and healthy controls. Relative expression of microRNAs was determined by S-Poly (T) Plus method in the tissue of 24 ARVC patients and 24 healthy controls. The miRNA levels were normalized to SNORD44 and represented in scatter plots. Data were shown as means ± SE, *p < 0.1 vs. healthy controls, **p < 0.01 vs. healthy controls, ***p < 0.001 vs. healthy controls.

**Figure 4:**
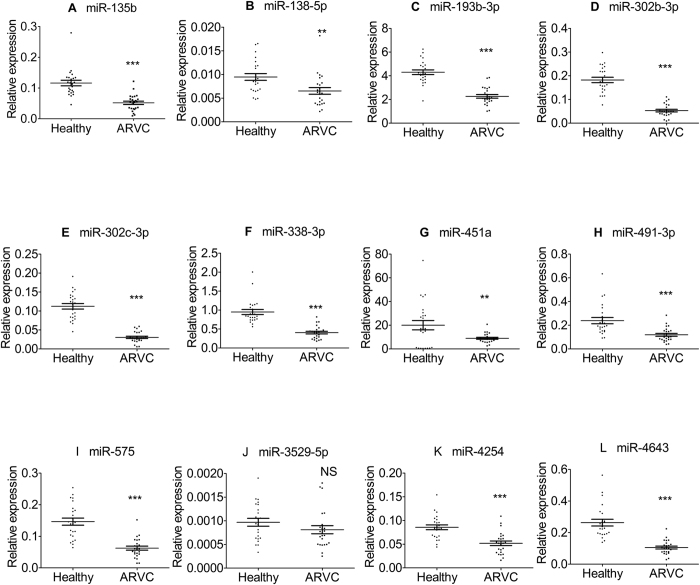
12 miRNAs were confirmed to be significantly down-regulated in ARVC patients compared with control group. The miRNA levels were normalized to SNORD44 and represented in scatter plots. Data were shown as means ± SE, *p < 0.1 vs. normal controls, **p < 0.01 vs. normal controls,***p < 0.001 vs. normal controls.

To determine the validity of candidate microRNAs, the relative expression levels of the 24 identified microRNAs were investigated individually ARVC heart samples and healthy controls. All the data were normalized by SNORD44 which was widely used as internal reference control. Similar to the initial screen, 12 microRNAs were all confirmed to be significantly up-regulated in each ARVC samples as compared to control samples (Fig. 3), 11 microRNAs were confirmed consistently down-regulated, while the expression of miR-3529-5P was not significantly altered, p = 0.3116 (Fig. 4).

### Receiver operating characteristic (ROC) curves analysis

**Figure 5:**
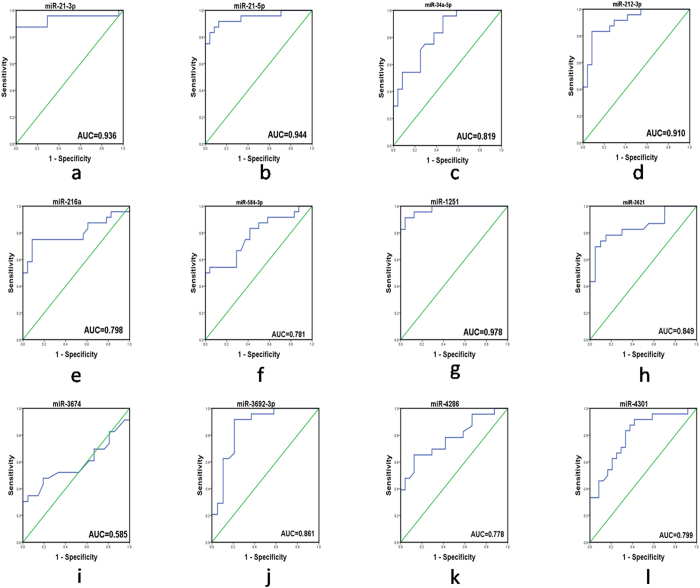
Receiver-operating characteristic (ROC) curve analyses of the 12 up-regulated miRNAs signature to discriminate ARVC patients from healthy controls. ROC analysis showed that AUC was 0.585 for miR-3674.

**Figure 6:**
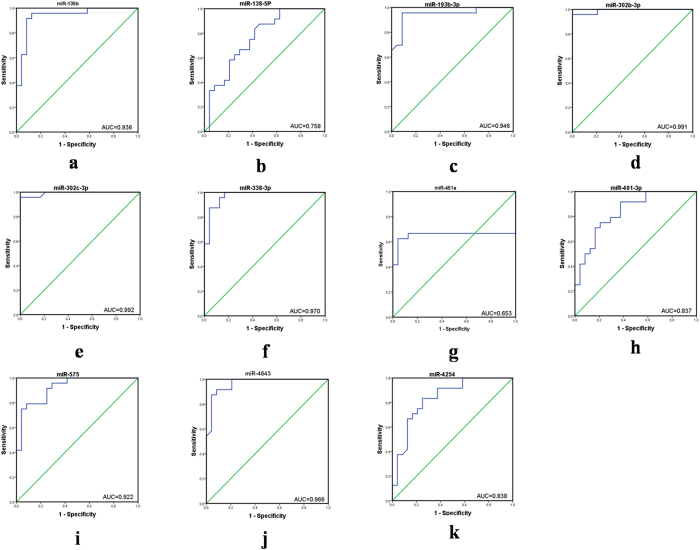
Receiver operating characteristic (ROC) curve analysis using 11 down-regulated microRNAs selected in large-scale validation and the miRNA panel classifier over patients with ARVC and healthy control subjects. ROC analysis showed that AUC was 0.653 for miR-451a.

To test whether differences of identified the 23 microRNAs could distinguish ARVC patients from healthy controls, we performed ROC curve analysis. The results showed that 11 of 12 significantly increased microRNAs had an optimal area value under the curve(AUC), among them, miR-1251, miR-21-3p, miR-21-5p, miR-212-3p and miR-34a-5p allowed for the discrimination between ARVC and healthy groups, corresponding to AUC of 0.978, 0.936, 0.944, 0.910 and 0.819 respectively ; however, miR-3674 was excluded for the AUC of it was 0.585 (Fig. 5). By contrast, 10 of 11 significantly decreased microRNAs got optimal AUC, the AUC of these microRNAs were 0.936, 0.758, 0.946, 0.991, 0.992, 0.970, 0.837, 0.922, 0.838, 0.966 for miR-135b, miR-138-5p, miR-193b-3p, miR-302b-3p, miR-302c-3p, miR-338-3p, miR-491-3P, miR-575, miR-4254 and miR-4643 respectively (Fig. 6). ROC analysis showed that AUC was 0.653, 0.585 for miR-451a and miR-3674, with a sensitivity of 56.5% and a specificity of 75%, suggesting that miR-451a and miR-3674 had no application value. The sensitivity, specificity and the cut off value of other microRNAs, corresponded to the best performance according to the respective ROC curve analyses were showed on Supplementary Table S2.

### Enrichment analysis and construction of miRNA-gene network

**Figure 7:**
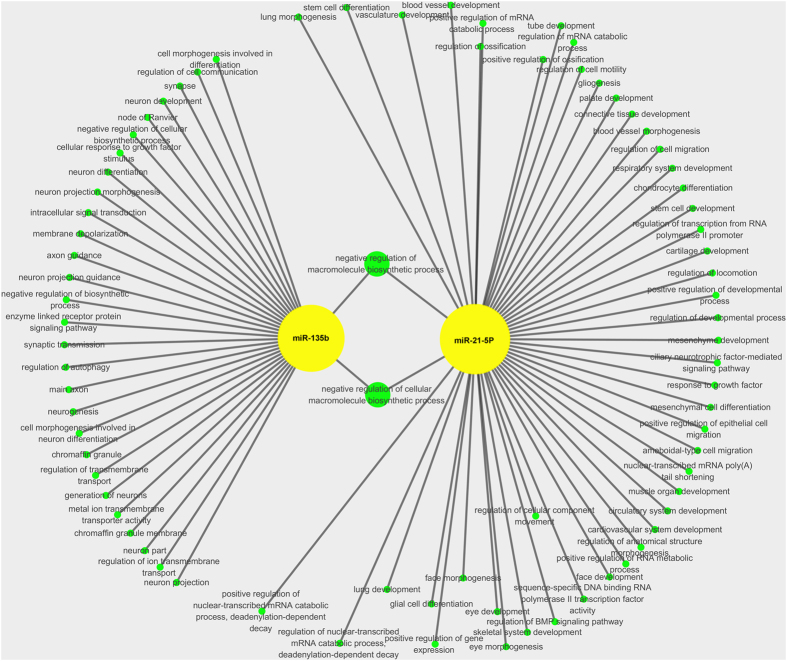
The miRNA-GO-network. Potential targets for miR-21-5p and miR-135b were searched from Targetscan (http://www.targetscan.org/). GO analysis were carried out with online software KOBAS 2.0(http://kobas.cbi.pku.edu.cn/home.do). Green circular nodes represent genes, and yellow circular nodes indicate miR-21-5p and miR-135b.

**Figure 8:**
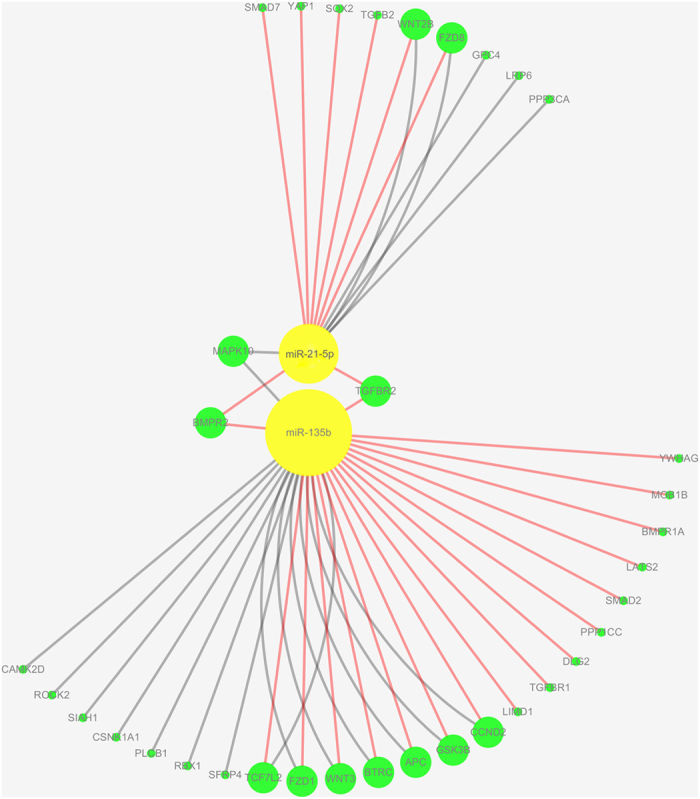
KEGG analysis was carried out with online software KOBAS 2.0 (http://kobas.cbi.pku.edu.cn/home.do). The miRNA-gene network indicated the relationship between miR-21-5p/miR-135b and Wnt/Hippo pathway was built using the software Cytoscape. Green circular nodes represent genes, and yellow circular nodes represent miR-21-5p or miR-135b. Red edges denoted the Hippo pathway, and grey edges indicated Wnt pathway.

Both Wnt and Hippo signaling play significant role in development of ARVC^9^,^12^. Among those microRNAs screened in our study, miR-21-5p and miR-135b have been reported to be involved in Wnt and Hippo pathway^28^,^29^. Therefore, we chose these two microRNAs for further analysis. Firstly, we searched for the potential targets of these 2 microRNAs from online software Targetscan (http://www.targetscan.org/). Gene ontology (GO) analysis for these targets was performed utilizing online software KOBAS 2.0 (http://kobas.cbi.pku.edu.cn/home.do), as shown in Fig. 7. KEGG pathway analysis was also analyzed with KOBAS 2.0, and miRNA-gene network involved in Wnt and Hippo pathway was built (Fig. 8). Two target genes BMPR2 and TGFBR2 were noticed, which were regulated by miR-21 and miR-135b in common. BMPR2 was highly expressed in heart and associated with adipogenesis^30^. TGFBR2 related to TGFB signal pathway that contributed to the extracellular matrix production. Our analysis results suggested that both miR-21 and miR-135b and their target genes BMPR2, TGFBR2 and genes related to Wnt and Hippo pathway in the coordinate regulation of pathology of ARVC.

## Discussion

In the present study, we aimed to evaluate the extensive genome-wide miRNA expression dysregulation in ARVC patients. Therefore, we first used a novel qRT-PCR method named the S-Poly(T) Plus, to assay the global microRNAs expression profile in a ARVC pooled RNA sample. The most important finding of this study was that 21 significantly changed microRNAs were identified independently in diseased myocardium, miRNA-gene network of miR-21-5p and miR-135b involved in Wnt and Hippo pathway was established.

MicroRNAs were involved in heart diseases such as: cardiac hypertrophy, heart failure, and acute myocardial infarction^21^. However, there were still no researches evaluating the global expression of microRNAs in ARVC heart tissue. Comprehensive analysis of miRNA expression between ARVC patients and healthy controls will help us to better understand the roles of microRNAs in the initiation and development of the disease as well as to find novel therapeutic targets for ARVC.

Previous numerous studies have demonstrated that microRNAs was essential to heart normal function, through a wide range of orchestrating biological process by post-transcriptionally regulating the expression of protein-coding genes^21^,^31^. Some researchers have demonstrated several microRNAs were drawn into myocardial fibrosis, cardiomyocytes apoptosis and regeneration^32^. In the present study, we also identified several microRNAs (e.g. miR-21-5p, miR-135b, miR-34a-5p, miR-212-3p, miR-302b-3p and miR-302c-3p) which proved by previous studies of heart disease with consistently aberrant expression in ARVC patients. Among these microRNAs, miR-21-5p, miR-34a and miR-212 have been shown to play an essential role in normal heart development and function in previous studies^33^,^34^,^35^.

Moreover, miR-21-5p showed abnormal expressions in cardiovascular diseases, including hypertrophic cardiomyopathy and fibrosis^36^, coronary diseases^37^,^38^, dilated cardiomyopathy^39^, heart failure^40^. In this work, miR-21-5p was one of the most significantly up-regulated microRNAs in ARVC patients, suggesting its materiality function in ARVC. It was also shown that increased miR-21-5p expression predominantly taking place in fibroblasts by pressure overload, miR-21-5p inhibition attenuated cardiomyocytes fibrosis and heart dysfunction through inhibiting Sprouty homologue 1 (Spry 1) and regulating the ERK-MAP kinase signaling pathway^36^.

To date, the molecular mechanisms for pathogenesis of ARVC mainly concentrate upon the Wnt and Hippo signaling pathway^41^. Molecular remodeling of desmosome disruption in ARVC perturb the key intracellular signaling pathway, the Hippo/YAP pathway, invoke the Hippo pathway and inhibit the canonical Wnt signaling, and then enhance adipogenesis in ARVC^9^,^12^. Some key genes such as large tumor suppressor kinases 1/2 (LATS1/2), and Yes-associated protein (YAP), STE20-like protein kinases 1/2 (MST1/2), transcription factor 7-like 2 (TCF7L2), Mob kinase activator 1 a/b (MOB1a/b) *et al*. play essential roles in these signaling pathway^42^,^43^. Previous studies have shown that miR-21-5p and miR-135b were involved in Wnt and Hippo signaling pathway, and regulated key target genes to regulate apoptosis, cell proliferation, angiogenesis, adipogenesis and fibro-genesis^29^,^44^,^45^.

The canonical Wnt and the Hippo pathways are partly regulated at the cell membrane and likely through the intercalated discs (IDs), and play crucial roles in development of ARVC^41^. Furthermore, we identify the putative targets of miR-21-5p and miR-135b and construct the network of these genes on Wnt and Hippo pathway. In order to explore the miR-21-5p, miR-135b target genes and their biological function, we explored target scan signatures using IPA, and constructed a miRNA-gene network which included miR-21-5p, miR-135b and pivotal target genes in Wnt and Hippo pathway. GO analysis indicated that two microRNAs may be involved with muscle organ development, circulatory system development, cardiovascular system development and cell proliferation. The mechanism of these microRNAs regulating the target genes in Wnt and Hippo pathway might be important and to needs further investigation in ARVC.

Recently, miR-34a was reported to reduce cardiomyocyte apoptosis in infarcted murine hearts and the insulin-like growth factor-1(IGF-1) anti-apoptotic effect was canceled when miR-34a was over expression^46^,^47^. A study showed that miR-34a was highly expressed in murine as early as one week postnatal and throughout the lifespan, so, it may be acritical factor of heart repair and regeneration of post-acute myocardial infarction in neonatal hearts, providing a novel way to adjust the adult cardiomyocytes^48^. Ahmetset al. have shown that miR-212 null mice could prevent the heart failure induced by pressure-overload, and directly target the FoxO3, an anti-hypertrophic and pro-autophagic transcription factor. Over expression of miR-212 was sufficient to induce hyper activation of pro-hypertrophic Calcineurin/NFAT signalling and impaired autophagy^49^. A new miRNA-cross-talk including miR-212 and miR-30a-3p, through suppressing significant endothelial genes such as Grb2 associated binder 1 (GAB1) and Sirtuin 1(SIRT1) finally culminating in impaired endothelial function^33^. The miR-302 cluster are essential in early embryonic development and somatic cell reprogramming^50^,^51^. Christienet al. showed that the miR-302 promoter was regulated by the Wnt/β-catenin signaling pathway through Tcf3, the only Tcf/Lef factor that bound to the miR-302 promoter^52^.

In summary, we identified the signatures of 21 microRNAs in the heart tissue of ARVC patients with precise quantification, miR-21-5p and miR-135b may play important roles in the regulation of genes in Wnt and Hippo signal pathway resulting in the manifestation and phenotype of ARVC. These microRNAs might reveal the potential pathophysiology mechanisms of ARVC, and might to be useful as therapeutic targets for ARVC. Although our results contributed to the new avenue of microRNAs in ARVC and provide the rationale for physiopathology of ARVC, certainly the exact mechanisms of these miRNA functions need to be validated in the future.

## Methods

### Ethics statement and tissue samples collection

The 24 control samples were derived from autopsies or donors with no history of heart disease who died in accidents. All ARVC participants gave informed written consent for this investigation, which was approved by the Institutional Ethical Review Board of Fuwai Hospital (Beijing, China). The investigation conforms to the principles outlined in the Declaration of Helsinki. Heart tissue samples of right ventricular were collected between the year of 2005 and 2014 confirmed using the Task Force criteria for ARVC^53^. All tissue samples used in the experiments were carefully snap-frozen in liquid nitrogen until total RNA was extracted.

### Pipeline in this study

**Figure 9:**
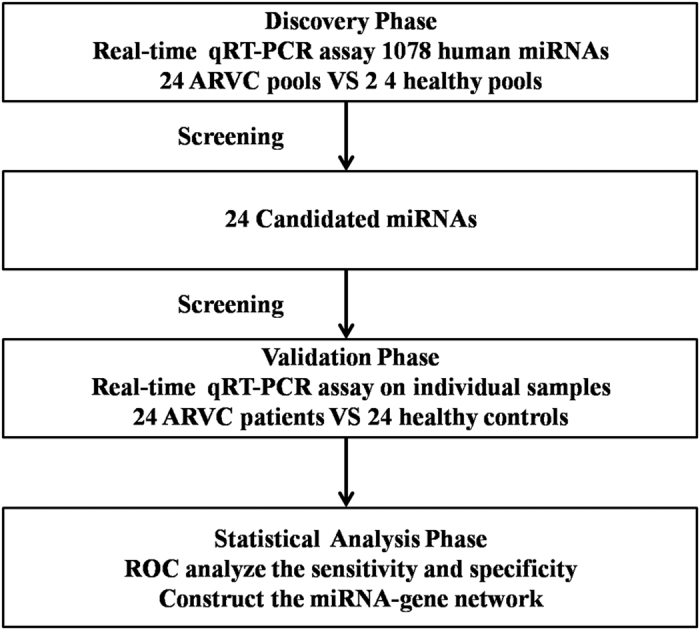
An optimized protocol for miRNA analysis using S-Poly (T) Plus qRT-PCR.

We designed a multiphase case-control study to investigate the expression of microRNAs in heart tissue (Fig. 9). In the initial screening phase, approximately 1078 microRNAs were divided into 154 groups, with each group included 7 microRNAs and SNORD44, a normalization control. All the microRNAs were assayed in two RNA-mixtures from 24 ARVC patients and 24 healthy controls using the S-Poly (T) Plus method. We chose those microRNAs with more than a 1.5-fold change in ARVC group compared to control group, these microRNAs were selected for further validation in each individual of 24 patients and 24 healthy controls.

### RNA extraction

Total RNA from each heart sample was extracted with RNAiso Plus (Takara biotechnology Co, Dalian, China) according to the manufacturer’s protocol. Briefly, 1 ml of RNAiso-Plus (TaKaRa, Dalian, China) was added to 100 mg of particulate sample which was grinded in liquid nitrogen and quickly mixed thoroughly. Then 200 μl of chloroform was added and mixed by vigorous shaking for 20 s. The samples were incubated for 5 min at room temperature and centrifuged at 12,000 g for 15 min at 4 °C. An equal volume (500 μl) of isopropyl alcohol was added into the mixture and incubated at −20 °C for 10 min followed by centrifugation at 13,500 g for 10 min at 4 °C. Then the final RNA was washed with 1 ml of 75% ethanol and eluted in 20 μl of RNase-free water. The concentration and purity of RNA solution were quantified by identifying absorbance at 260–280 nm using the NanoDrop 2000c Spectrophotometer (Thermo Fisher Scientific, Wilmington, DE) and stored at −80 °C until analysis.

### Polyadenylation and reverse transcription

In the S-Poly (T) Plus method, polyadenylation and reverse transcription were performed in a single step with an optimized buffer. In briefly, the following components were added to 10 μl of reaction mixture: 100 ng of total RNAs from samples, 2.5 μl of 4 × reaction buffer mix containing ATP and dNTPs, 1 μl of 0.5 μM RT primer and 1 μl of polyA/RT enzyme mix (with 0.8 units of Poly(A) polymerase and 100 units of MMLV High Performance Reverse Transcriptase). The reaction was incubated at 37 °C for 30 min, 42 °C for 30 min, and then terminated at 75 °C for 5 min.

### Real-time PCR

For comparison, 0.5 μl cDNA for S-Poly(T) was used in real-time PCR reaction for tissue samples to allow an equal amount of RNA inputs. The reaction mixture (20 μl) consisted of 4 μl 5 × qPCR Reaction Buffer, 0.5 unit hotstar Taq Polymerase (Geneup, Shenzhen, China), 0.4 μlc 10 μM forward primer, 0.4 μl 10 μM universal reverse primer, 0.5 μl 10 μM universal Taqman probe, and 0.2 μl 100 × ROX reference dye. Real-time PCR was carried out on ABI StepOne Plus thermal cycler at the following conditions: 95 °C for 3 min, followed by 40 cycles of 95 °C for 10 s and 60 °C for 30 s. Reactions were performed in triplicate

### Target prediction and network

In order to identify miR-21-5p, miR-135b on Wnt pathway and Hippo pathway putative targets, the software Targetscan (http://www.targetscan.org/) and KOBAS 2.0 (http://kobas.cbi.pku.edu.cn/home.do) were used. According to the interactions between microRNAs and the intersected genes of pathway, we built a miRNA-gene-network which illustrates network pathway analysis and the key regulatory functions of the identified microRNAs, with software Cytoscape.

### Statistical analysis

Relative quantities of microRNAs were calculated using the 2^^−ΔCt^ method with SNORD44 as a normalization control. Two-tailed Student’s test was used for statistical analysis and data was shown as means ± SE (standard error).Receiver operating characteristic (ROC) curve analysis was made to calculate the area under the curve (AUC) of individual miRNA, AUC was used to evaluate the specificity and sensitivity of microRNAs for differentiating between ARVC patients group and healthy controls group, an optimal area value under the curve (AUC) >0.70 was used to predicted probabilities. A difference of *P* < 0.05 (two-sided) was considered statistically significant. All statistical analyses were performed by SPSS 17.0 software (SPSS, Chicago, IL, USA) or Graphpad Prism (version 5.0; Graphpad software).

## Additional Information

**How to cite this article**: Zhang, H. *et al*. Profiling of differentially expressed microRNAs in arrhythmogenic right ventricular cardiomyopathy. *Sci. Rep.* **6**, 28101; doi: 10.1038/srep28101 (2016).

## Supplementary Material

Supplementary Information

## Figures and Tables

**Table 1:** Clinical characteristics of ARVC patients.

ARVC Patients	n	Value
Age (yrs)	24	36 ± 13
Men	16	66%
Family history of ARVC	0	0%
Family history of sudden death	3	12.50%
Palpitation	23	95.80%
Dizziness	10	41.66%
syncope	6	25.50%
Atrial fibrillation	9	37.50%
VT-LBBB morphology	8	33.33%
Non-sustained tachycardia	15	62.50%
VF	1	4.10%
ICD implant	5	20.83%
Echocardiography		
LVEF		28.52 ± 8.55
RV dilatation		38.52 ± 10.60
Pharmacological therapy		
Beta-blockers	21	87.50%
ACEIs/AIIRAs	23	95.80%
Diuretics	22	91.70%
Digoxin	14	58.33%
Aspirin/Clopidogrel	15	62.50%
Warfarin	9	37.50%
New York Heart Association functional class		
II	3	12.50%
III	7	29.16%
IV	14	58.33%
