# Supplementary material for: Profiling of differentially expressed microRNAs in arrhythmogenic right ventricular cardiomyopathy
Source: Sci Rep. 2016 Jun 16;6:28101. doi: 10.1038/srep28101 (PMC4910108; doi:10.1038/srep28101)
Supplement: Supplementary Information [file srep28101-s1.pdf]

Profiling of differentially expressed microRNAs in arrhythmogenic right ventricular cardiomyopathy

Hongliang Zhang[1,2,3], Shenghua Liu[1],Tianwei Dong[2] , Jun Yang[2], Yuanyuan Xie[1], Yike Wu[3], Kang Kang[4], shengshou Hu[1], Deming Gou[3#], Yingjie Wei[1#]

Supplementary Information

Supplementary table S1　Primers and probe sequences used in S-Poly(T) plus method.

Supplementary table S2　The area under the curve (AUC) of individual miRNA.

Supplementary table S3　The scores of GO terms of miR-21-5p and miR-135b.

Supplementary table S1　Primers and probe sequences for S-Poly(T)　method.

miRNA		Sequence (5'-3')	Forward primer	RT primer
>hsa-miR-21-3p	MIMAT0004494	CAACACCAGUCGAUGGGCUGU	TCGGCAACACCAGTCGATG	GTGCAGGGTCCGAGGTCAGAGCCACCTGGGCAATTTTTTTTTTTACAGCC
>hsa-miR-21-5p	MIMAT0000076	UAGCUUAUCAGACUGAUGUUGA	TTCGGTAGCTTATCAGACTGA	GTGCAGGGTCCGAGGTCAGAGCCACCTGGGCAATTTTTTTTTTTCAACAT
>hsa-miR-34a-5p	MIMAT0000255	UGGCAGUGUCUUAGCUGGUUGU	CGGTGGCAGTGTCTTAGCT	GTGCAGGGTCCGAGGTCAGAGCCACCTGGGCAATTTTTTTTTTTACAACC
>hsa-miR-212-3p	MIMAT0000269	UAACAGUCUCCAGUCACGGCC	TTCGGTAACAGTCTCCAGTC	GTGCAGGGTCCGAGGTCAGAGCCACCTGGGCAATTTTTTTTTTTGGCCGT
>hsa-miR-216a	MIMAT0000273	UAAUCUCAGCUGGCAACUGUGA	TCGGTAATCTCAGCTGGCAA	GTGCAGGGTCCGAGGTCAGAGCCACCTGGGCAATTTTTTTTTTTCACAGT
>hsa-miR-584-3p	MIMAT0022708	UCAGUUCCAGGCCAACCAGGCU	TGGTCAGTTCCAGGCCAAC	GTGCAGGGTCCGAGGTCAGAGCCACCTGGGCAATTTTTTTTTTTAGCCTG
>hsa-miR-1251	MIMAT0005903	ACUCUAGCUGCCAAAGGCGCU	TTCGGACTCTAGCTGCCAAA	GTGCAGGGTCCGAGGTCAGAGCCACCTGGGCAATTTTTTTTTTTAGCGCC
>hsa-miR-3621	MIMAT0018002	CGCGGGUCGGGGUCUGCAGG	GGCGCGGGTCGGGGTC	GTGCAGGGTCCGAGGTCAGAGCCACCTGGGCAATTTTTTTTTTTCCTGCA
>hsa-miR-3674	MIMAT0018097	AUUGUAGAACCUAAGAUUGGCC	GTCGGATTGTAGAACCTAAGA	GTGCAGGGTCCGAGGTCAGAGCCACCTGGGCAATTTTTTTTTTTGGCCAA
>hsa-miR-3692-3p	MIMAT0018122	GUUCCACACUGACACUGCAGAAGU	GGTTCCACACTGACACTGC	GTGCAGGGTCCGAGGTCAGAGCCACCTGGGCAATTTTTTTTTTTACTTCT
>hsa-miR-4286	MIMAT0016916	ACCCCACUCCUGGUACC	TGGTCGGACCCCACTCCT	GTGCAGGGTCCGAGGTCAGAGCCACCTGGGCAATTTTTTTTTTTGGTACC
>hsa-miR-4301	MIMAT0016850	UCCCACUACUUCACUUGUGA	GTCGGTCCCACTACTTCAC	GTGCAGGGTCCGAGGTCAGAGCCACCTGGGCAATTTTTTTTTTTCACAAG
>hsa-miR-135b	MIMAT0000758	UAUGGCUUUUCAUUCCUAUGUGA	TCGGTATGGCTTTTCATTCCT	GTGCAGGGTCCGAGGTCAGAGCCACCTGGGCAATTTTTTTTTTTCACATA
>hsa-miR-138-5p	MIMAT0000430	AGCUGGUGUUGUGAAUCAGGCCG	TGGAGCTGGTGTTGTGAATC	GTGCAGGGTCCGAGGTCAGAGCCACCTGGGCAATTTTTTTTTTTCGGCCT
>hsa-miR-193b-3p	MIMAT0002819	AACUGGCCCUCAAAGUCCCGCU	CGGAACTGGCCCTCAAAGT	GTGCAGGGTCCGAGGTCAGAGCCACCTGGGCAATTTTTTTTTTTAGCGGG
>hsa-miR-302b-3p	MIMAT0000715	UAAGUGCUUCCAUGUUUUAGUAG	TCGGTAAGTGCTTCCATGTTT	GTGCAGGGTCCGAGGTCAGAGCCACCTGGGCAATTTTTTTTTTTCTACTA
>hsa-miR-302c-3p	MIMAT0000717	UAAGUGCUUCCAUGUUUCAGUGG	TCGGTAAGTGCTTCCATGTTT	GTGCAGGGTCCGAGGTCAGAGCCACCTGGGCAATTTTTTTTTTTCCACTG
>hsa-miR-338-3p	MIMAT0000763	UCCAGCAUCAGUGAUUUUGUUG	TCGGTCCAGCATCAGTGATT	GTGCAGGGTCCGAGGTCAGAGCCACCTGGGCAATTTTTTTTTTTCAACAA
>hsa-miR-451a	MIMAT0001631	AAACCGUUACCAUUACUGAGUU	TTCGGAAACCGTTACCATTAC	GTGCAGGGTCCGAGGTCAGAGCCACCTGGGCAATTTTTTTTTTTAACTCA
>hsa-miR-491-3p	MIMAT0004765	CUUAUGCAAGAUUCCCUUCUAC	TCGGCTTATGCAAGATTCCC	GTGCAGGGTCCGAGGTCAGAGCCACCTGGGCAATTTTTTTTTTTGTAGAA
>hsa-miR-575	MIMAT0003240	GAGCCAGUUGGACAGGAGC	GTCGGGAGCCAGTTGGAC	GTGCAGGGTCCGAGGTCAGAGCCACCTGGGCAATTTTTTTTTTTGCTCCT
>hsa-miR-3529-5p	MIMAT0019828	AGGUAGACUGGGAUUUGUUGUU	TCGGAGGTAGACTGGGATTT	GTGCAGGGTCCGAGGTCAGAGCCACCTGGGCAATTTTTTTTTTTAACAAC
>hsa-miR-4254	MIMAT0016884	GCCUGGAGCUACUCCACCAUCUC	GGCCTGGAGCTACTCCAC	GTGCAGGGTCCGAGGTCAGAGCCACCTGGGCAATTTTTTTTTTTGAGATG
>hsa-miR-4643	MIMAT0019703	GACACAUGACCAUAAAUGCUAA	TTCGGGACACATGACCATAAA	GTGCAGGGTCCGAGGTCAGAGCCACCTGGGCAATTTTTTTTTTTAGCATT
SNORD-44		CCTGGATGATGATAAGCAAATGCTGAC TGAACATGAAGGTCTTAATTAGCTCTAA CTGACT	TGGCCTGGATGATGATAAGCA	GTGCAGGGTCCGAGGTCAGAGCCACCTGGGCAATTTTTTTTTTTAGTCAG
Universal reverse primer		CAGTGCAGGGTCCGAGGT		
Universal Taqman probe		56-FAM/CAGAGCCAC/ZEN/CTGGGCAATTT/3IABkFQ		

Supplementary table S2 the area under the curve (AUC) of individual miRNA .

miRNA name	AUC	P value	Cut off	sensitivity(%)	Specificity(%)
miR-21-3p	0.936	< 0.001	0.0255	87.5	100
miR-21-5p	0.944	< 0.001	9.497	87.5	91.7
miR-34a-5p	0.836	< 0.001	0.942	95.8	70.8
miR-212-3p	0.91	< 0.001	0.0751	83.3	92.7
miR-216a	0.8	< 0.001	0.027	75	91.3
miR-584-3p	0.786	0.001	0.254	42	95.7
miR-1251	0.978	< 0.001	0.497	91.3	95.8
miR-3621	0.849	< 0.001	1.183	69.6	95
miR-3692-3p	0.861	< 0.001	0.049	91.7	97.9
miR-4286	0.778	0.001	58.49	65.2	87.5
miR-4301	0.799	< 0.001	6.442	91.7	54.2
miR-135b	0.932	< 0.001	0.073	95.2	85.7
miR-138-5p	0.758	0.002	0.006	87.5	54.2
miR-193b-3p	0.946	< 0.001	3.085	95.7	91.3
miR-302b-3p	0.991	< 0.001	0.104	95.8	95.8
miR-302c-3p	0.992	< 0.001	0.051	95.8	91.7
miR-338-3p	0.996	< 0.001	0.709	87.5	95.2
miR-491-3P	0.837	< 0.001	0.123	91.7	95.200
miR-575	0.922	< 0.001	0.099	79.2	91.7
miR-4254	0.838	< 0.001	0.062	83.3	75
miR-4643	0.966	< 0.001	0.159	91.7	91.7

Supplementary table S3 The scores of GO terms of miR-21-5p and miR-135b.

miRNA	ID	Corrected P-Value	GO_term
miR-21-5p	GO:0060425	0.006025082	lung morphogenesis
miR-21-5p	GO:0048863	0.006025082	stem cell differentiation
miR-21-5p	GO:0001944	0.006797634	vasculature development
miR-21-5p	GO:0001568	0.006797634	blood vessel development
miR-21-5p	GO:0061014	0.006864386	positive regulation of mRNA catabolic process
miR-21-5p	GO:0030278	0.007161766	regulation of ossification
miR-21-5p	GO:0035295	0.008845766	tube development
miR-21-5p	GO:0061013	0.008845766	regulation of mRNA catabolic process
miR-21-5p	GO:0045778	0.008845766	positive regulation of ossification
miR-21-5p	GO:2000145	0.008845766	regulation of cell motility
miR-21-5p	GO:0042063	0.008845766	gliogenesis
miR-21-5p	GO:0060021	0.00970205	palate development
miR-21-5p	GO:0061448	0.010819458	connective tissue development
miR-21-5p	GO:0048514	0.011050044	blood vessel morphogenesis
miR-21-5p	GO:0030334	0.011944962	regulation of cell migration
miR-21-5p	GO:0060541	0.012527326	respiratory system development
miR-21-5p	GO:0002062	0.012527326	chondrocyte differentiation
miR-21-5p	GO:0048864	0.012615338	stem cell development
miR-21-5p	GO:0006357	0.012615338	regulation of transcription from RNA polymerase II promoter
miR-21-5p	GO:0051216	0.012615338	cartilage development
miR-21-5p	GO:0040012	0.012615338	regulation of locomotion
miR-21-5p	GO:0051094	0.012615338	positive regulation of developmental process
miR-21-5p	GO:0050793	0.012615338	regulation of developmental process
miR-21-5p	GO:2000113	0.015442092	negative regulation of cellular macromolecule biosynthetic process
miR-21-5p	GO:0060485	0.015740946	mesenchyme development
miR-21-5p	GO:0070120	0.017406701	ciliary neurotrophic factor-mediated signaling pathway
miR-21-5p	GO:0070848	0.017680308	response to growth factor
miR-21-5p	GO:0048762	0.017680308	mesenchymal cell differentiation
miR-21-5p	GO:0010634	0.017680308	positive regulation of epithelial cell migration
miR-21-5p	GO:0001667	0.017680308	ameboidal-type cell migration
miR-21-5p	GO:0000289	0.017680308	nuclear-transcribed mRNA poly(A) tail shortening
miR-21-5p	GO:0010558	0.017680308	negative regulation of macromolecule biosynthetic process
miR-21-5p	GO:0007517	0.017680308	muscle organ development
miR-21-5p	GO:0072359	0.017680308	circulatory system development
miR-21-5p	GO:0072358	0.017680308	cardiovascular system development
miR-21-5p	GO:0022603	0.017680308	regulation of anatomical structure morphogenesis
miR-21-5p	GO:0051254	0.018364844	positive regulation of RNA metabolic process
miR-21-5p	GO:0060324	0.01959787	face development
miR-21-5p	GO:0000981	0.01959787	sequence-specific DNA binding RNA polymerase II transcription factor activity
miR-21-5p	GO:0051270	0.019890116	regulation of cellular component movement

miR-21-5p	GO:0030510	0.019890116	regulation of BMP signaling pathway
miR-21-5p	GO:0001501	0.020759783	skeletal system development
miR-21-5p	GO:0001654	0.020759783	eye development
miR-21-5p	GO:0048592	0.020759783	eye morphogenesis
miR-21-5p	GO:0060325	0.021126654	face morphogenesis
miR-21-5p	GO:0010628	0.021126654	positive regulation of gene expression
miR-21-5p	GO:0010001	0.021126654	glial cell differentiation
miR-21-5p	GO:0030324	0.021126654	lung development
miR-21-5p	GO:1900151	0.021126654	regulation of nuclear-transcribed mRNA catabolic process, deadenylation-dependent decay
miR-21-5p	GO:1900153	0.021126654	positive regulation of nuclear-transcribed mRNA catabolic process, deadenylation-dependent decay
miR-135b	GO:0043005	0.207449408	neuron projection
miR-135b	GO:0034765	0.207449408	regulation of ion transmembrane transport
miR-135b	GO:0097458	0.207449408	neuron part
miR-135b	GO:0042584	0.207449408	chromaffin granule membrane
miR-135b	GO:0046873	0.207449408	metal ion transmembrane transporter activity
miR-135b	GO:0048699	0.207449408	generation of neurons
miR-135b	GO:0034762	0.207449408	regulation of transmembrane transport
miR-135b	GO:0048667	0.207449408	cell morphogenesis involved in neuron differentiation
miR-135b	GO:0042583	0.207449408	chromaffin granule
miR-135b	GO:0022008	0.207449408	neurogenesis
miR-135b	GO:0010558	0.220615975	negative regulation of macromolecule biosynthetic process
miR-135b	GO:0044304	0.220615975	main axon
miR-135b	GO:0007167	0.220615975	enzyme linked receptor protein signaling pathway
miR-135b	GO:0010506	0.220615975	regulation of autophagy
miR-135b	GO:0007268	0.220615975	synaptic transmission
miR-135b	GO:0009890	0.220615975	negative regulation of biosynthetic process
miR-135b	GO:0097485	0.220615975	neuron projection guidance
miR-135b	GO:0007411	0.220615975	axon guidance
miR-135b	GO:0051899	0.220615975	membrane depolarization
miR-135b	GO:0030182	0.220615975	neuron differentiation
miR-135b	GO:0048812	0.220615975	neuron projection morphogenesis
miR-135b	GO:0071363	0.220615975	cellular response to growth factor stimulus
miR-135b	GO:0031327	0.220615975	negative regulation of cellular biosynthetic process
miR-135b	GO:0048666	0.237688267	neuron development
miR-135b	GO:0033268	0.237688267	node of Ranvier
miR-135b	GO:0045202	0.240472611	synapse
miR-135b	GO:2000113	0.240472611	negative regulation of cellular macromolecule biosynthetic process
miR-135b	GO:0035556	0.248522008	intracellular signal transduction
miR-135b	GO:0010646	0.248522008	regulation of cell communication
miR-135b	GO:0000904	0.26182242	cell morphogenesis involved in differentiation
miR-135b	GO:0048666	0.237688267	neuron development
